# Coenzyme Q10 Regulates Antioxidative Stress and Autophagy in Acute Myocardial Ischemia-Reperfusion Injury

**DOI:** 10.1155/2017/9863181

**Published:** 2017-11-19

**Authors:** Shao Liang, Zhang Ping, Jin Ge

**Affiliations:** ^1^Department of Cardiology, The Second Affiliated Hospital and Yuying Children's Hospital of Wenzhou Medical University, Wenzhou, Zhejiang Province, China; ^2^Department of Geriatrics & Neurology, The Second Affiliated Hospital and Yuying Children's Hospital of Wenzhou Medical University, Wenzhou, Zhejiang Province, China

## Abstract

**Background:**

Oxidative stress and autophagy both play key roles in continuous cardiomyocyte death and cardiac dysfunction after reperfusion therapy for acute myocardial ischemia-reperfusion injury. Coenzyme Q10 (CQ10), which is a fat-soluble quinone antioxidant, is involved in the pathophysiological processes of neurodegenerative diseases, cancer, diabetes, heart failure, and other diseases. Our objective was to determine if, and by what mechanism, CQ10 can ameliorate acute myocardial ischemia-reperfusion injury and improve heart function.

**Methods and Results:**

Fat-soluble CQ10 in soybean oil solvent was preconditioned in rats with acute myocardial ischemia-reperfusion injury by intraperitoneal injection. Oxidant and antioxidant levels were compared between the preconditioned and control groups. Autophagy was measured by Western blotting analysis of autophagy proteins. Proapoptotic proteins and immunofluorescence were used to assess cell apoptosis. Infarct size was determined by triphenyl tetrazolium chloride (TTC) staining and Evans blue staining and visualized myocardial pathology by tissue staining. Finally, we assessed cardiac function by electrocardiography (ECG) and hemodynamics.

**Conclusions:**

This study reveals that CQ10 preconditioning regulates antioxidant levels and the oxidant balance, enhances autophagy, reduces myocardial apoptosis and death, and improves cardiac function in rats with acute ischemia-reperfusion injury. These results imply that CQ10 protects against acute myocardial ischemia-reperfusion injury via the antioxidative stress and autophagy pathways.

## 1. Introduction

Percutaneous coronary intervention is a principal therapeutic approach for the treatment of acute myocardial infarction, which has threatened the life and health of millions of people in recent years [1]. However, some patients still suffer from continuous myocardial cell death, further deterioration of cardiac function, and lower long-term survival rates [2]. The potential mechanisms of ischemia-reperfusion injury are complex and may include distal and/or collateral microcirculation, acute oxygen stress injury, mitochondrial damage, and inflammation [3–6].

Oxidative stress is particularly important in ischemia-reperfusion injury through cellular homeostasis, mitosis, cell differentiation, and intracellular signal transduction, which result in cell membrane rupture, swelling, or death [7]. Disruption of the balance between oxidants and antioxidants is serious in acute ischemia-reperfusion injury [8]. Oxidative stress also acts as a signal to induce autophagy in acute ischemia-reperfusion injury [9]. Autophagy is an intracellular defense and stress regulation mechanism that works by degrading damaged organelles and denaturing long-lived proteins and nucleic acids to provide raw materials and energy for cell surviving from injury, apoptosis, and death [10, 11]. Lower oxidative stress is very important for the activation and induction of autophagy; however, some oxidants also modify and inactivate Atg4 (autophagy-related protein 4), Atg5, LC-3 (microtubule-associated protein light chain-3), and other proteins. LC-3 is a necessary component of the autophagosome membrane and becomes involved after activation by Atg7 and Atg3; Atg5 couples with Atg12 to help LC-3 locate on the autophagosome membrane [12–14]. Beclin-1 is a protein that interacts with either Bcl-2 or PI3k class III and plays a critical role in the regulation of both autophagy and cell death [15]. And p62, as an autophagosome-degradation marker, is a multifunctional protein located throughout the cell and involved in many signal transduction pathway [16]. Autophagy is closely linked to apoptosis. Caspase-3 is a frequently activated death protease, catalyzing the specific cleavage of many key cellular proteins, and will be fully active under normal and apoptotic cell conditions. Caspase-3 is activated in the apoptotic cell by both extrinsic (death ligand) and intrinsic (mitochondrial) pathways [17, 18]. p53 protein plays a role in apoptosis, genomic stability, and inhibition of angiogenesis and initiates apoptosis [19]. They are essential proteins in the autophagy and/or apoptosis process.

Coenzyme Q10 (CQ10) is a fat-soluble quinone antioxidant with a structure that is similar to those of vitamin K and vitamin E. Recent research has identified CQ10 as an effective antioxidant for the prevention of oxidative damage, and it breaks down macromolecules to prevent inflammatory responses [20, 21]. Emerging research has shown that CQ10 is able to stabilize mitochondrial calcium-dependent ion channels and reduces cell energy depletion [22, 23]. However, the role of exogenous CQ10 in myocardial ischemic disease is still debatable [24–26].

Therefore, in this study, we aimed to determine if, and by what mechanism, CQ10 can ameliorate acute myocardial ischemia-reperfusion injury and improve heart function.

## 2. Materials and Methods

### 2.1. Animals and Drug Treatment

Adult male Sprague Dawley (SD) rats (body weight: 250 ± 10 g) were provided by the animal center of the Medical College of Xi'an Jiaotong University. Throughout the experiment, the animals were given ad libitum access to tap water.

The rats were divided into two groups: an acute myocardial ischemia-reperfusion group (AMI/R) and an acute myocardial ischemia-reperfusion with CQ10 group (CQ10 + AMI/R). The two groups were further divided into groups according to the following time points: sham, 2 h (2 h after reperfusion), 24 h, and 72 h.

In the AMI/R group, soybean oil solvent was intraperitoneally injected; in the CQ10 + AMI/R group, 5 mL fat-soluble CQ10 (6 mg·kg^−1^·mL^−1^) (number 24893170, Sigma, China) in soybean oil solvent was intraperitoneally injected [20]. CQ10 was consequent administered 3 days before acute ischemia and following reperfusion until the end of the experiment.

### 2.2. Acute Myocardial Ischemia-Reperfusion Model

The AMI/R rat model was established as described previously [27]. SD rats were anesthetized with 2% pentobarbital (20 mg·kg^−1^) and connected to animal electrocardiography (ECG) equipment (GE Medical, Milwaukee, WI, USA). Before exposing the heart, the rats were ventilated by positive pressure using a small animal respirator (Harvard Apparatus Rodent Respirator, Harvard, USA) after tracheal intubation. The left anterior descending (LAD) coronary artery was consecutively ligated for 45 min before reperfusion injury, whereas in the sham group, the LAD coronary artery was not ligated but was merely wound with string. A benzylpenicillin sodium (400000 U/kg) injection was administered through intraperitoneal once a day for 3 days to prevent infection. Successful AMI/R operations were confirmed by ST segment-characterized ECG.

During the whole operation, the tension of the limbs, the heart rate, and autonomous respiration were monitored to assess the level of anesthesia. A mixture of pentobarbital and feed was supplied to minimize the pain caused by the procedure. As described in a previous study [23], a single injection was sufficient to keep the rats under anesthesia for the entire procedure.

At the end of the experiment, 2% pentobarbital (100 mg·kg^−1^) was injected into the abdomen of each rat to induce euthanasia before analysis of the AMI/R model. Eight rats in which the induction of remodeling had been successful were identified in each group. There were totally 134 rats (128 total samples) in this research for avoiding unsuccessful construction and that the same one could not be used in various detecting methods, such as echocardiography, hemodynamics, myocardial staining, histopathology, and immunohistochemistry.

### 2.3. Echocardiography and Hemodynamic Assessment of Cardiac Function

The structure and heart function of each subject were evaluated by the length of the 10S probe shaft and the use of an M-type two-dimensional ultrasound instrument (Sequoia 512, Siemens AG, Germany), respectively. Three consecutive cardiac cycles were captured from at least six subjects from each group by digital image analysis software. Subsequently, a sidearm of the aortic cannula was inserted into the left ventricle from the right carotid artery and was connected to a pressure transducer (Millar Instruments, Houston, Texas, USA). The organism function experimental system (type number BL-420F, Texas, USA) was used to amplify the signal and analyze the data. Myocardial tissue was preserved for the subsequent step.

### 2.4. Western Blotting Analysis

Equal amounts of proteins from each sample were separated with Any-kD sodium dodecyl sulfate (SDS) precast gel. After blocking with 5% nonfat milk, polyvinylidene fluoride membranes were hybridized with the following antibodies: monoclonal beclin-1 antibody at 1 : 1000 dilution, monoclonal Atg5 antibody at 1 : 1000 dilution, monoclonal LC-3A/B antibody at 1 : 1000 dilution, monoclonal p53 antibody at 1 : 1000 dilution, and monoclonal caspase-3 antibody at 1 : 1000 dilution. The membranes were subsequently hybridized with the corresponding secondary antibodies. *β*-Actin proteins were used as loading and internal controls. The data are shown as the grayscale ratio of the target protein to *β*-actin.

### 2.5. Myocardial Tissue Staining

Each myocardial tissue sample was cut transversely. Myocardial pathology was observed by hematoxylin and eosin (HE) staining and Masson trichrome staining. Myocardial ischemic risk areas were determined as described previously [28]. To delineate the ischemic area-at-risk, 1% Evans blue (1 mL) was injected following the occlusion of the coronary artery at the level of LAD ligation. The whole heart was then removed and transected parallel to the atrioventricular groove at the center of the infarct area in 1% triphenyl tetrazolium chloride (TTC) solution for 10 min. There were at least six rats in each group and three sections per rat. The observer was blinded to the animal treatment groups when evaluating myocardial infarct sizes.

For immunofluorescence staining, 4′,6-diamidino-2-phenylindole (DAPI), Bax antibody, Bak antibody, and the corresponding fluorescent secondary antibodies were used. Immunofluorescence staining was quantified by Image-Pro Plus software. In the AMI/R rats, the DAPI, Bax, or Bak fluorescence density values of the sham group were set to 100 standard density; other groups were represented by fluorescence density values relative to the standard density.

### 2.6. Measurement of Oxidants and Antioxidants

Heart tissue was weighed and homogenized in ice-cold normal saline. Heart homogenate was mixed with an equal volume of cold Tris-ethylenediaminetetraacetic acid (EDTA). The separated cytosolic fraction was used for the subsequent measurement.

Oxidant levels were measured using a thiobarbituric acid reactive substances (TBARS) assay kit (Cayman Chemical Company, Ann Arbor, USA).

The levels of the antioxidants glutathione peroxidase (GPx), glutathione reductase (GR), glutathione (GSH), and superoxide dismutase (SOD) were measured in each group according to the instructions provided by the manufacturers of the GPx assay kit (Jining, Shanghai, China), the GR assay kit (Yueyan Biological Technology Co. Ltd., Shanghai, China), the GSH assay kit (Jiancheng, Nanjing, China), and the SOD assay kit (Dojindo, Shanghai, China), respectively.

### 2.7. Detection of Cellular Energy Supply

The ATPlite Luminescence Assay System (PerkinElmer, USA) was used to evaluate cellular energy supply according to manufacturer's instructions. Data are shown as ATP concentrations relative to the ATPlite Assay System standard sample.

### 2.8. Statistical Analysis

The random variables follow a normal distribution in this analysis. All data are presented as mean ± standard, and *p* < 0.05 was considered statistically significant. Analysis of variance (ANOVA) was used to examine differences between the two groups, followed by either the least significant difference (LSD) test or the Games–Howell test according to the homogeneity of variance.

### 2.9. Compliance with Ethical Standards

The animal protocol was approved by the Xi'an Jiaotong University laboratory animal ethics review board (number A-2012-975SL), and the animal procedures conformed with the guidelines provided by Directive 2010/63/EU of the European Parliament on the protection of animals used for scientific purposes.

## 3. Results

### 3.1. CQ10 Reduced Oxidative Stress in Rats with Acute Myocardial Ischemia-Reperfusion Injury

Because CQ10 is a well-known antioxidant agent, we demonstrated that preconditioning with CQ10 significantly increased antioxidant levels of GPx, GR, SOD, and GSH (*p* < 0.05, *n* = 6) and decreased oxidant TBARS levels (*p* < 0.05, *n* = 6) in rats with acute myocardial ischemia-reperfusion injury (Table 1).

### 3.2. CQ10 Enhanced Autophagy in Rats with Acute Myocardial Ischemia-Reperfusion Injury

**Figure 1:**
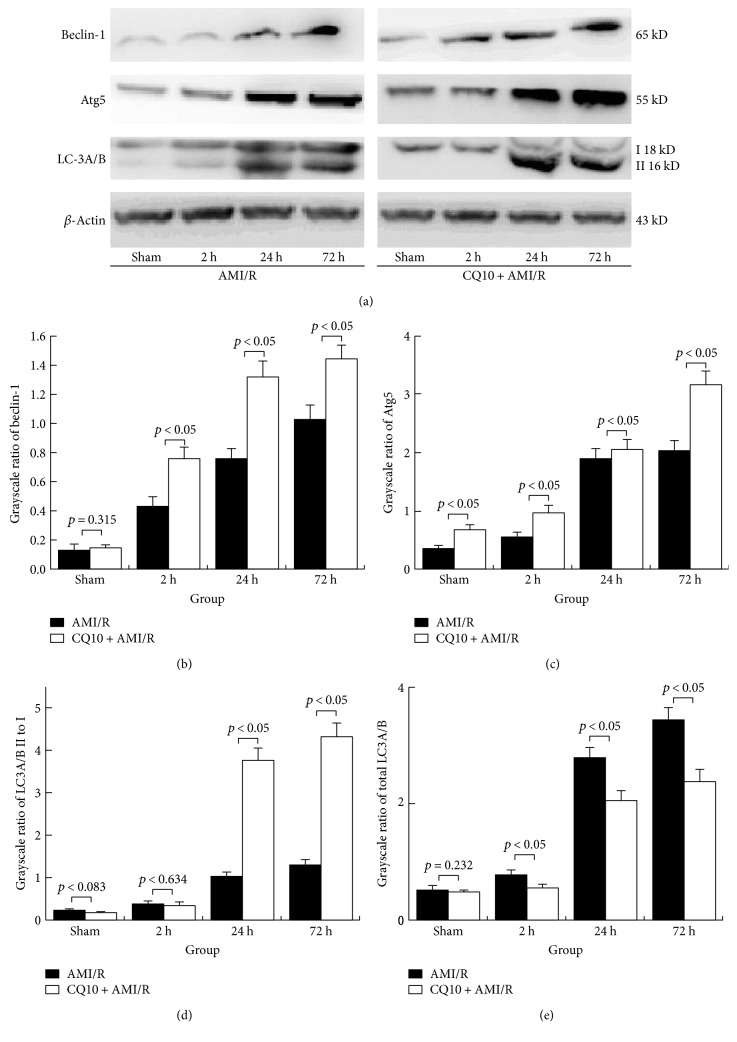
CQ10 enhanced autophagy in rats with acute myocardial ischemia-reperfusion injury. (a) The grayscale of beclin-1, Atg5 LC-3A/B, and *β*-actin was detected by Western blot. (b and c) CQ10 increased autophagy beginning protein beclin-1 and Atg5 expression at same time point. (d and e) CQ10 increased autophagy activity and improve the transformation of LC-3A/B II to I. *p* < 0.05 was considered statistically significant, *n* = 6.

**Figure 2:**
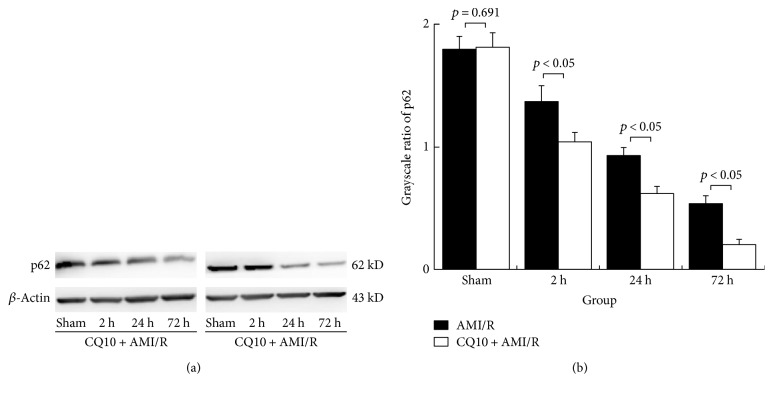
CQ10 decreased p62 protein expression (as grayscale ratio of p62 to *β*-actin) in rats with acute myocardial ischemia-reperfusion injury. (a) The grayscale of autophagy-negative protein p62 expression was detected by Western blot. (b) CQ10 decreases p62 protein expression at same time point. *p* < 0.05 was considered statistically significant, *n* = 6.

We used Western blotting detection to demonstrate that CQ10 noticeably increased the levels of the positively associated autophagy proteins beclin-1 and Atg5 at the same time points of acute myocardial ischemia-reperfusion injury (*p* < 0.05, *n* = 6). Furthermore, the ratio of LC-3II to LC-3I as a standard of autophagy activity was clearly increased by CQ10 at the same time points as acute myocardial ischemia-reperfusion injury (Figure 1; *p* < 0.05, *n* = 6). However, the total LC-3 and negatively associated autophagy protein p62 levels were significantly reduced in the CQ10 group compared with the AMI/R group at the same time points in (Figure 2; *p* < 0.05, *n* = 6).

### 3.3. CQ10 Reduced Proapoptotic Proteins in Rats with Acute Myocardial Ischemia-Reperfusion Injury

**Figure 3:**
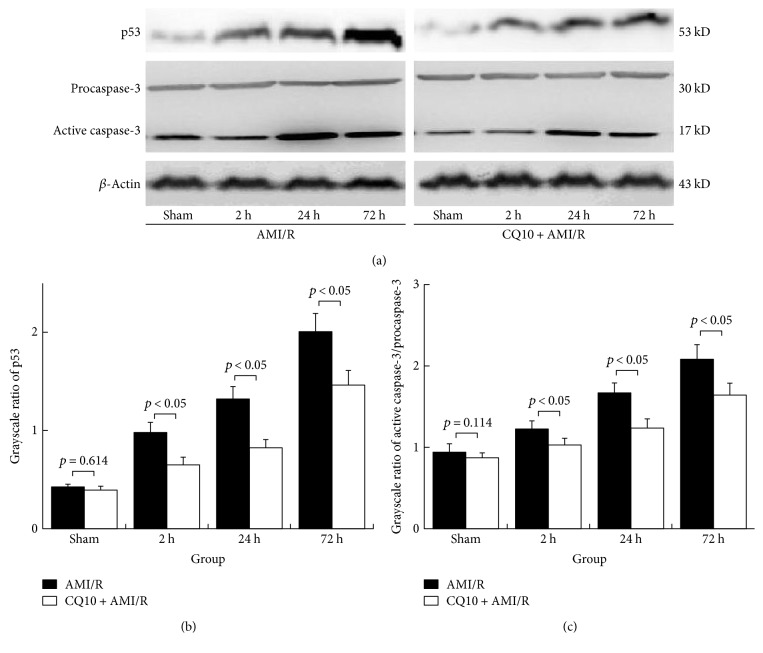
(a) p53 and caspase-3 expression (as grayscale ratio of p53 or caspase-3 to *β*-actin) in rats with acute myocardial ischemia-reperfusion injury was shown. (b and c) CQ10 decreases apoptosis-associated protein expression of p53 and caspase-3. Caspase-3 activity was shown as a grayscale ratio of active caspase-3 to procaspase-3. *p* < 0.05 was considered statistically significant, *n* = 6.

We also proved that CQ10 reduced proapoptotic protein p53, and the ratio of active caspase-3 to procaspase-3 was significantly higher at 2 h, 24 h, and 72 h in rats with acute myocardial ischemia-reperfusion injury than in the control rats (Figure 3; *p* < 0.05, *n* = 6).

**Figure 4:**
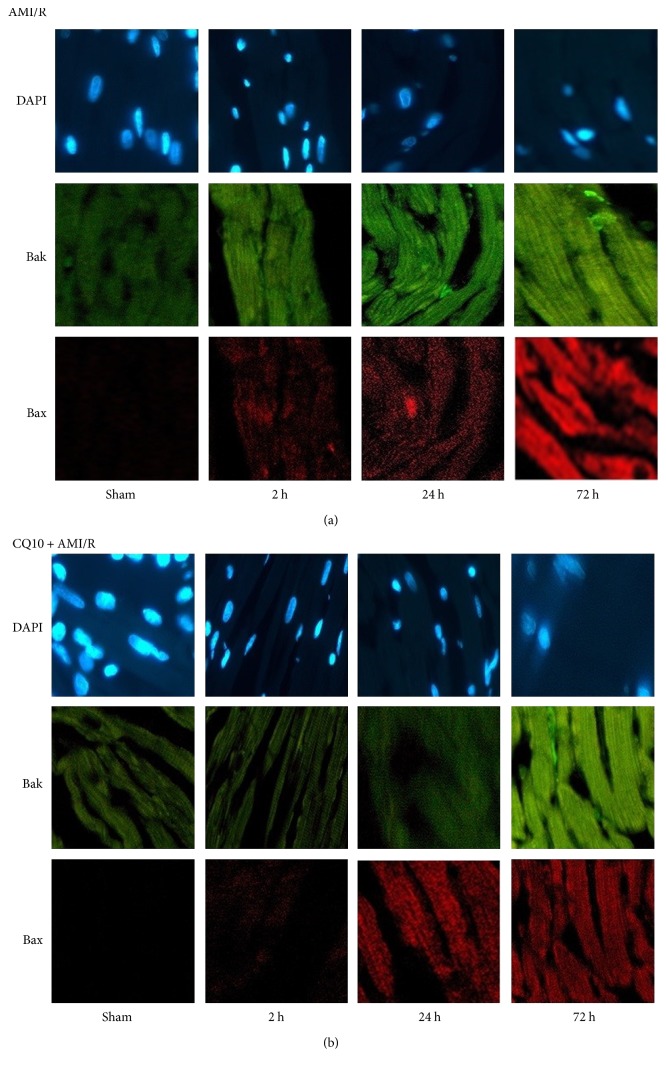
DAPI/Bak/Bax immunofluorescence staining in rats with acute myocardial ischemia-reperfusion injury (×400). (a) DAPI/Bak/Bax immunofluorescence staining in different time of AMI/R group. (b) DAPI/Bak/Bax immunofluorescence staining in different time of AMI/R + CQ10 group.

**Figure 5:**
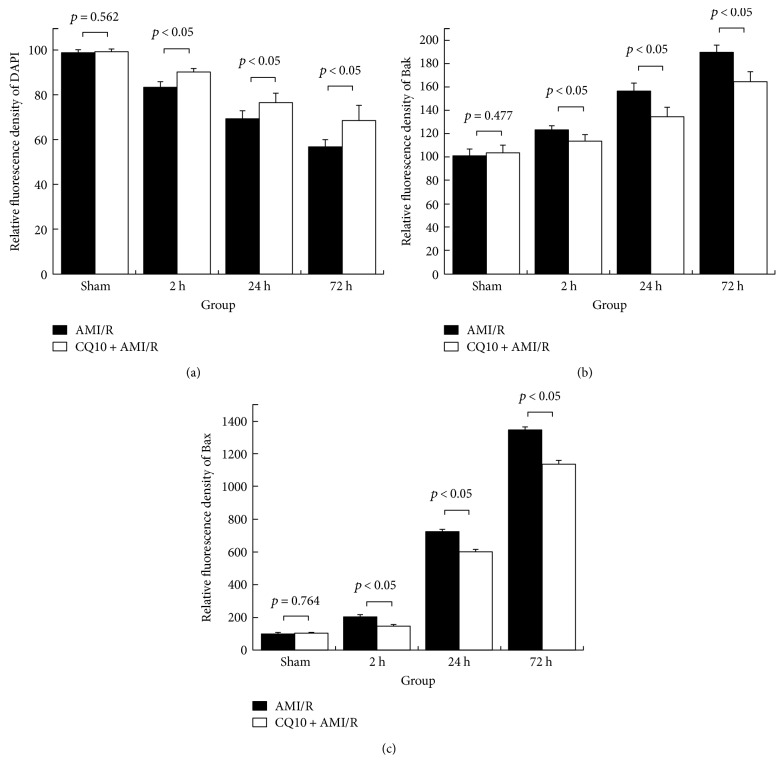
Relative fluorescence density of DAPI/Bak/Bax in rats with acute myocardial ischemia-reperfusion injury. Sham group's DAPI, Bax, or Bak fluorescence density was set as 100 standard density. Other groups' DAPI, Bax, or Bak fluorescence density was present as relative fluorescence density compared with sham group. Six samples were selected from each group, and three horizons were observed in the ischemic penumbra of each sample (×400). (a) CQ10 increases DAPI fluorescence density. (b) CQ10 decreases Bax fluorescence density. (c) CQ10 decreases Bak fluorescence density. *p* < 0.05 was considered statistically significant.

DAPI fluorescence revealed the number of intact nuclei, but did not show myocardial necrosis due to DNA and RNA condensation, fission, or burst. Compared with the AMI/R group, there were more myocardial nuclei in the CQ10 group at the same time points. Bak/Bax fluorescence expression was stronger in the CQ10 group than in the AMI/R group at the same time points (Figures 4 and 5; *p* < 0.05).

### 3.4. CQ10 Preserved Myocardial Activity in Rats with Acute Myocardial Ischemia-Reperfusion Injury

**Figure 6:**
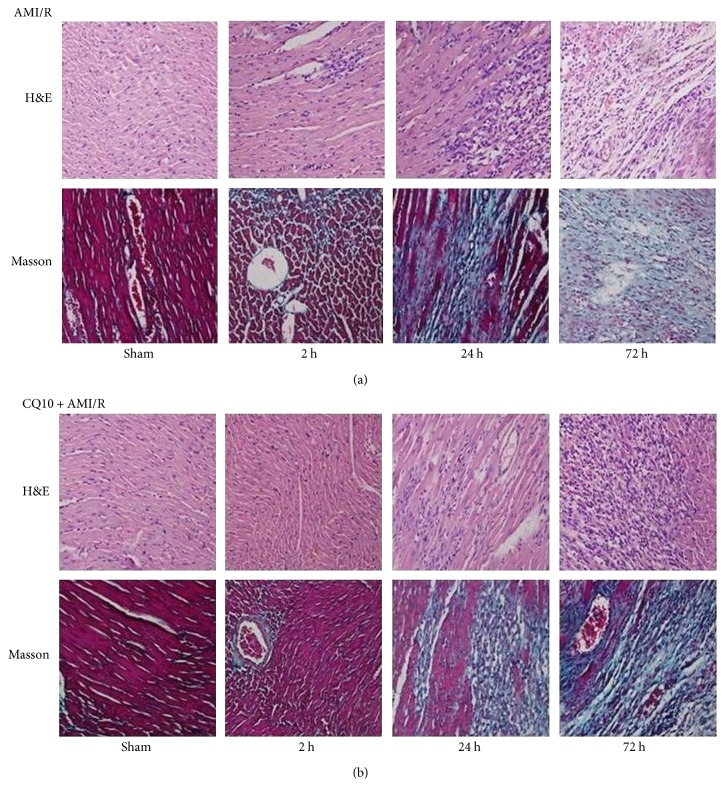
CQ10 could delay myocardial fibrosis, denaturation, and necrosis in rats with acute myocardial ischemia-reperfusion injury by H&E and Masson staining (×100). (a) H&E and Masson staining in different time of AMI/R group. (b) H&E and Masson staining in different time of AMI/R + CQ10 group.

HE and Masson staining facilitated the visualization of cardiac structural disorder, myocardial cell denaturation and necrosis, and fibrous scar tissue formation in rats with acute myocardial ischemia-reperfusion injury. However, in the ischemic penumbra of the CQ10 group, these phenomena were not as serious as in the AMI/R group (Figure 6).

**Figure 7:**
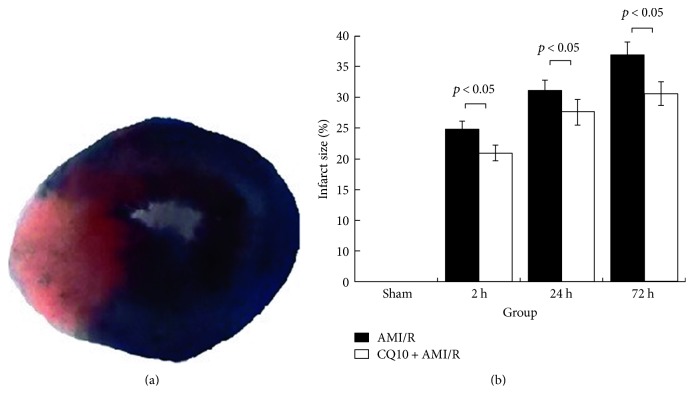
CQ10 could reduce infarct size (as a percentage of the ischemic area-at-risk) in rats with acute myocardial ischemia-reperfusion injury by TTC staining. (a) Infarct area appeared khaki, whereas the viable myocardium was red by TTC staining. (b) CQ10 could reduce the percentage of the ischemic area-at-risk. *p* < 0.05 was considered statistically significant, *n* = 6.

Infarct size was estimated as a percentage of the ischemic area-at-risk. At 2 h, 24 h, and 72 h following acute myocardial ischemia-reperfusion injury, CQ10 significantly reduced infarct size (Figure 7; *p* < 0.05, *n* = 6).

### 3.5. CQ10 Improved Cardiac Function in Rats with Acute Myocardial Ischemia-Reperfusion Injury

We used echocardiography and hemodynamic analysis to investigate cardiac function during acute myocardial ischemia-reperfusion injury. There was no difference between the two groups with regard to the following parameters: diastolic interventricular septal wall thickness (IVSd), systole interventricular septal wall thickness (IVS), diastolic left ventricular internal dimension (LVIDd), systole left ventricular internal dimension (LVID), and systole volume (SV), which represented cardiac structure and size at the same average heart rate (*p* > 0.05, *n* = 6). From 2 h after reperfusion, CQ10 preconditioning improved cardiac systolic and diastolic functions (left ventricular ejection fraction (EF), left ventricular systolic pressure (LVSP), and left ventricular systolic pressure (LVEDP), the maximum change rate of left ventricular pressure rise (+dp/dtmax), and the maximum change rate of left ventricular pressure fall (−dp/dtmax) compared with the AMI/R group (Table 2; *p* < 0.05, *n* = 6)).

### 3.6. CQ10 Increased Direct Energy Supply in Rats with Acute Myocardial Ischemia-Reperfusion Injury

**Figure 8:**
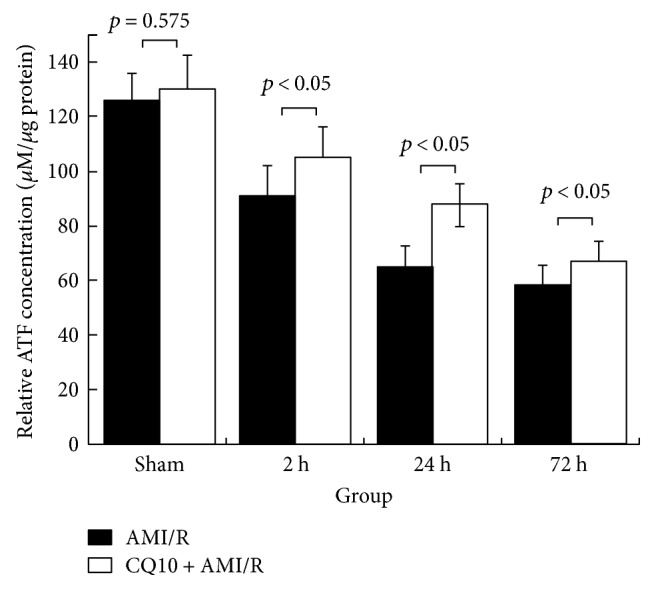
CQ10 increased relative ATP concentration in rats with acute myocardial ischemia-reperfusion injury. Date was shown as relative ATP concentration according to ATPlite Assay System standard sample. *p* < 0.05 was considered statistically significant, *n* = 6.

Finally, the relative ATP concentration, which is representative of direct energy supply, was increased by CQ10 during acute myocardial ischemia-reperfusion injury (Figure 8).

## 4. Discussion

It has become increasingly clear that the level of oxidants is greatly increased in the postischemic heart, and oxidants play a critical role in postischemic injury. Lipid peroxidation, induced by oxygen radicals, results in cell membrane breakdown, which leads to inhibition of the sodium-potassium ATPase, cell swelling, and mitochondrial dysfunction [6]. As previously reported, CQ10 plays a key role in the mitochondrial respiratory chain by preventing oxidative stress injury to the mitochondrial membrane [29, 30]. It is worth noting that there is a synergistic effect between Bax and Bak on mitochondrial fragmentation and perforation, cell apoptosis, and programmed cell death. In our research, both the balance between antioxidants (GPx, GR, SOD, and GSH) and oxidants and the levels of Bax and Bak were regulated by CQ10. Furthermore, the ATP concentration increased after CQ10 preconditioning for direct energy supply.

Autophagy is a widespread self-ingesting phenomenon that normally takes place in the biosphere at a low level. It is considered to be a protection mechanism that acts by degrading impaired proteins and/or organelles. It ensures that the cells survive with adequate supplies of materials and energy through recycling under most conditions of stress, such as acute ischemia and reperfusion, chemical toxicity, and oxidation [31–33]. However, high levels of oxidants are responsible for the inactivity of Atg5, beclin-1, and other autophagy-associated proteins [12, 14, 34]. Other studies have also proved that preconditioning with autophagy-inducing agents protects myocardial cells after acute ischemia-reperfusion [34]. In our study, Atg5, beclin-1, and the ratio of LC-3II to LC3-I, which represent autophagy activity, are increased after acute ischemia-reperfusion injury with CQ10 preconditioning. The study also demonstrates that CQ10 reduces total LC-3 levels and p62.

Emerging evidence suggests interactions among the crucial proteins of autophagy and apoptosis as follows: beclin-1 binding to Bcl-2 family, caspase-3 cleaving beclin-1, [35], p53 promoting the proapoptotic proteins and inhibiting Bcl-2, and p53 posttranscriptionally downregulating LC-3 and controlling autophagic flux [36]. In our study, apoptosis protein levels are reduced by CQ10 preconditioning. Both proapoptosis protein and apoptosis protein of Bcl-2 family have taken important roles in cell apoptosis process. Cell intact nuclei were stained by DAPI. Bak and Bax were located in the mitochondrial outer membrane by immunofluorescence staining. Bax and Bak have a collaborative effect to increase the mitochondrial outer membrane permeability and aggravate cell death. As previously informed, p53 and caspase-3 are also wildly proved proapoptosis effect in many stress condition.

In all, antioxidative function, energy survival, and autophagy activation are the major causes of infarct size reduction and cardiac function improvement after acute ischemia-reperfusion injury with CQ10 preconditioning. But timely reperfusion of the acute ischemic myocardium is essential for myocardial salvage. A number of mechanisms have been proposed to explain the mediation of reperfusion injury. These include cellular calcium loading, endothelium cell swelling, impaired vascular relaxation, and the action of oxygen radicals [3–6]. The impact of CQ10 is actually restricted to delaying the changes after ischemia-reperfusion injury, not abolishing anything [15, 24]. In clinical situations, the outcomes for acute coronary syndrome patients who are suffering from ischemia-reperfusion are influenced by many unpredictable events, such as myocardial stunning, coronary vascular spasms, or lethal arrhythmia. Further research is required [24, 25].

For some limitation, this study just focuses on short period of outcome after ischemia-reperfusion injury. In order to sufficiently evaluate whether CQ10 preconditioning reduces or only delays the detrimental effect of ischemia-reperfusion injury, a research designed for different doses of CQ10 and a long of outcome after ischemia-reperfusion injury is needed.

In summary, we have established that CQ10 preconditioning regulates the balance of antioxidants and oxidants, enhances autophagy levels and activity, reduces myocardial apoptosis, and improves cardiac function in rats with acute ischemia-reperfusion injury. This study provides a new therapeutic target for acute ischemia-reperfusion injury and enriches our understanding of the clinical application of CQ10.

## Figures and Tables

**Table 1:** Effect of CQ10 on the change of SOD, GSH, TBARS, GPx, and GR in rat myocardial tissue with acute myocardial ischemia-reperfusion injury.

	AMI/R				CQ10 + AMI/R			
	Sham	2 h	24 h	72 h	Sham	2 h	24 h	72 h
SOD (U/mg protein)	26.89 ± 2.42	20.96 ± 2.23	15.13 ± 1.89	10.76 ± 1.92	27.29 ± 2.14	22.25 ± 2.35^∗^	18.63 ± 2.41^&^	14.31 ± 1.98^#^
GSH (*μ*mol/mg protein)	5.82 ± 0.54	4.42 ± 0.28	3.13 ± 0.33	2.64 ± 0.32	5.98 ± 0.37	5.38 ± 0.43^∗^	4.27 ± 0.37^&^	3.47 ± 0.41^#^
TBARS (nmol/mg protein)	1.33 ± 0.09	2.74 ± 0.14	3.94 ± 0.29	5.38 ± 0.37	1.29 ± 0.12	2.38 ± 0.19^∗^	3.19 ± 0.31^&^	4.57 ± 0.42^#^
GPx (*μ*mol/protein)	7.41 ± 0.64	5.43 ± 0.52	3.12 ± 0.33	2.14 ± 0.23	7.38 ± 0.56	6.11 ± 0.44^∗^	4.57 ± 0.42^&^	3.25 ± 0.31^#^
GR (*μ*mol/protein)	5.14 ± 0.31	3.28 ± 0.34	2.11 ± 0.27	1.89 ± 0.21	5.23 ± 0.29	4.56 ± 0.38^∗^	3.75 ± 0.42^&^	3.01 ± 0.44^#^

CQ10 increased antioxidation (SOD, GSH, GPx, and GR) and reduced oxidation (TBARS) in rats with acute myocardial ischemia-reperfusion injury at same time point. SOD: superoxide dismutase; GSH: glutathione; TBARS: thiobarbituric acid reactive substances; GPx: glutathione peroxidase; GR: glutathione reductase. ^∗^*p* < 0.05 compared with 2 h in AMI/R group, *n* = 6; ^&^*p* < 0.05 compared with 24 h in AMI/R group, *n* = 6; ^#^*p* < 0.05 compared with 72 h in AMI/R group, *n* = 6.

**Table 2:** The change of cardiac function.

	AMI/R				CQ10 + AMI/R			
	Sham	2 h	24 h	72 h	Sham	2 h	24 h	72 h
HR (bpm)	395.26 ± 35.78	396.14 ± 42.74	402.20 ± 42.12	418.54 ± 39.56	412.70 ± 39.34	397.24 ± 36.24	409.20 ± 38.92	392.87 ± 42.13
IVSd (mm)	1.64 ± 0.27	1.58 ± 0.34	1.62 ± 0.31	1.61 ± 0.32	1.64 ± 0.34	1.72 ± 0.28	1.65 ± 0.41	1.68 ± 0.39
IVS (mm)	2.56 ± 0.37	2.30 ± 0.44	2.55 ± 0.81	2.49 ± 0.44	2.67 ± 0.13	2.47 ± 0.58	2.55 ± 0.18	2.43 ± 0.21
LVIDd (mm)	4.11 ± 0.94	4.18 ± 1.48	4.15 ± 0.67	4.21 ± 0.67	4.15 ± 0.19	3.97 ± 0.16	4.05 ± 0.45	4.14 ± 0.42
LVID (mm)	2.45 ± 0.57	2.50 ± 0.95	2.53 ± 0.79	2.43 ± 0.89	2.53 ± 0.18	2.57 ± 0.12	2.45 ± 0.27	2.49 ± 0.38
SV (cm^3^)	0.25 ± 0.08	0.24 ± 0.05	0.23 ± 0.04	0.23 ± 0.06	0.22 ± 0.07	0.24 ± 0.06	0.20 ± 0.08	0.23 ± 0.10
FS (%)	53.17 ± 5.76	54.74 ± 1.87	55.45 ± 9.63	52.37 ± 4.13	52.12 ± 4.82	50.13 ± 2.07	54.17 ± 7.88	51.21 ± 4.37
EF (%)	93.25 ± 3.14	80.23 ± 7.12	70.26 ± 5.26	59.12 ± 5.81	92.14 ± 2.65	85.42 ± 5.27^∗^	79.56 ± 5.36^∗^	67.12 ± 6.18^∗^
LVSP (mmHg)	84.25 ± 12.85	74.67 ± 10.15	65.84 ± 9.84	61.84 ± 10.04	84.42 ± 12.55	79.45 ± 10.25^∗^	71.44 ± 14.13^∗^	67.82 ± 13.32^∗^
LVEDP (mmHg)	2.16 ± 0.09	2.53 ± 0.08	2.86 ± 0.12	3.42 ± 0.07	2.19 ± 0.14	2.33 ± 0.09^∗^	2.64 ± 0.17^∗^	2.97 ± 0.15^∗^
+dp/dtmax (mmHg/ms)	3.79 ± 0.12	3.37 ± 0.06	2.18 ± 0.07	1.84 ± 0.08	3.80 ± 0.08	3.49 ± 0.06	2.88 ± 0.09^∗^	2.25 ± 0.12^∗^
−dp/dtmax (mmHg/ms)	3.02 ± 0.06	2.56 ± 0.48	2.37 ± 0.54	1.57 ± 0.54	2.92 ± 0.08	2.75 ± 0.04^∗^	2.49 ± 0.09^∗^	1.89 ± 0.09^∗^

CQ10 improved cardiac function but did not change cardiac structure in rats with acute myocardial ischemia-reperfusion injury at same time point. IVSd: diastolic interventricular septal wall thickness; IVS: systole inter-ventricular septal wall thickness; LVIDd: diastolic left ventricular internal dimension; LVID: systole left ventricular internal dimension; EF: left ventricular ejection fraction; SV: systole volume; FS: left ventricular fractional shortening; LVSP: left ventricular systolic pressure; LVEDP: left ventricular end-diastolic pressure; +dp/dtmax: the maximum change rate of left ventricular pressure rise; −dp/dtmax: the maximum change rate of left ventricular pressure fall; AMI/R: acute myocardial ischemia/reperfusion. ^∗^*p* < 0.05 was considered statistically significant; *n* = 6.
